# Systematic Review of Human and Animal Evidence on the Role of Buckwheat Consumption on Gastrointestinal Health

**DOI:** 10.3390/nu15010001

**Published:** 2022-12-20

**Authors:** Ezra Valido, Jivko Stoyanov, Frida Gorreja, Stevan Stojic, Christa Niehot, Jessica Kiefte-de Jong, Erand Llanaj, Taulant Muka, Marija Glisic

**Affiliations:** 1 Swiss Paraplegic Research, 6207 Nottwil, Switzerland; 2 Department of Health Sciences, University of Lucerne, 6003 Lucerne, Switzerland; 3 Institute of Social and Preventive Medicine (ISPM), University of Bern, 3012 Bern, Switzerland; 4 Department of Microbiology and Immunology, Institute of Biomedicine, University of Gothenburg, S-405 30 Gothenburg, Sweden; 5 Literature Searches Support, 3000 GA Dordrecht, The Netherlands; 6 Department of Public Health and Primary Care, Health Campus The Hague, Leiden University Medical Center, 2300 RC Leiden, The Netherlands; 7 ELKH-DE Public Health Research Group of the Hungarian Academy of Sciences, Department of Public Health and Epidemiology, Faculty of Medicine, University of Debrecen, 4032 Debrecen, Hungary; 8 Department of Molecular Epidemiology, German Institute of Human Nutrition Potsdam-Rehbruecke, 14558 Nuthetal, Germany

**Keywords:** buckwheat, Tartary buckwheat, gastrointestinal symptoms, microbiome

## Abstract

Background: Buckwheat is a commonly cultivated crop with growing evidence that it is beneficial to gastrointestinal (GI) health. This systematic review summarizes the role of buckwheat in modifying GI health outcomes and microbiomes. Methods: Four medical databases and Google Scholar were systematically searched. Clinical trials, observational studies, animal in vivo, and in vitro studies with human and animal GI-derived samples were included. Results: There were 32 studies (one randomized controlled trial [RCT], one non-randomized trial, 3 observational, 9 in vitro, and 18 animal in vivo studies) included. In preclinical studies, buckwheat extracts were observed to have cytotoxic potential against human-derived GI cancer cell lines. Animals fed with buckwheat had lower GI mucosal inflammation, higher alpha diversity in the GI microbiome, and higher levels of fecal short-chain fatty acids. Human evidence studies and clinical trials were limited and predominantly of moderate risk of bias. The majority of in vitro studies with GI-derived samples and in vivo studies were reliable without restrictions in study design. Conclusion: In vivo and in vitro studies show that buckwheat may have potential GI benefits due to its anti-oxidant and anti-inflammatory potential; however, human evidence remains limited, and its impact on health in humans remains to be elucidated in future trials.

## 1. Introduction

Common buckwheat (*Fagopyrum esculentum*) and Tartary buckwheat (*F. tataricum*) are cultivated crops in various parts of the world. Buckwheat, the plant, and the seed, is a nutritious food source with high protein content and rich in fiber, polyphenols, flavonoids, and minerals and is often consumed as an alternative to cereals and other starchy foods [1,2,3,4,5]. The presence of these bioactive compounds has made buckwheat linked to potential health benefits due to anti-oxidant, anti-hypertensive, anti-inflammatory, and anti-diabetic effects [6,7].

The presence of several phyto-actives, being gluten-free, and low in fermentable oligosaccharide, disaccharide, monosaccharide, and polyols (FODMAP) [8], makes buckwheat an attractive substitute for individuals with a sensitive gastrointestinal (GI) tract, including those with gluten-sensitivity. To support this recommendation and to understand the role of buckwheat on GI health, we need to review the evidence. Furthermore, the role of buckwheat consumption on the GI microbiome has to be taken into consideration. The GI microbiome is known to be the key to the maintenance of GI tract health [9,10]. Diet can modulate the GI microbiome to either health-promoting communities or to dysbiosis that could lead to possible disease in the GI tract or contribute to other related pathologies [10]. Grains, such as oats, have been seen to increase the fermentation and production of healthy GI-promoting molecules such as short-chain fatty acids (SCFAs) and improve intestinal barrier function in humans [11].

To date, there have been no systematic reviews looking into the role of buckwheat consumption on GI health and especially on the GI microbiome and GI outcomes. Recent reviews have focused on the characterization of bioactive compounds present in the grain, while others have looked at cardiovascular health outcomes after buckwheat consumption [1,2,3,4,5,12,13,14]. This systematic review aims to summarize the human evidence exploring the association between habitual buckwheat consumption or the effect of buckwheat interventions on GI health and the GI microbiome. Further, to understand biological pathways involved in improved GI health, we aim to include pre-clinical in vivo or in vitro studies using either human or animal material. Moreover, we aim to critically assess the methodological quality of evidence, identify research gaps in the literature, and provide directions for further research.

## 2. Methods

### 2.1. Data Sources and Search Strategy

This review followed the guidance provided by Muka et al. [15], the Preferred Reporting Items for Systematic Reviews and Meta-Analyses Statement (PRISMA) guidelines [16], and the Prisma-S extension to the PRISMA Statement for Reporting Literature Searches in Systematic Reviews [17] (Supplemental Appendix SI). This review’s protocol was not publicly registered, but the protocol is attached as Supplemental Appendix SII. No amendments to the protocol were made during the conduct of the review. EMBASE (Embase.com), MEDLINE via Ovid, Cochrane Central (Wiley), and Web of Science (Clarivate) were searched from the date of inception until 17 January 2022. A single search string query was used to search the databases for reproducibility and adaptability. An additional search was performed in Google Scholar, and the 200 most relevant references were downloaded using Publish or Perish software [18]. The search included terms related to buckwheat and its scientific names and coupled with terms that relate to GI conditions, outcomes, and symptoms. The detailed search strategy can be seen in the protocol. In addition, reference lists of included studies were manually searched to identify additional relevant articles. The results were deduplicated using the Bramer/Erasmus MC method in EndNote [19]. No authors or subject experts were contacted.

### 2.2. Study Selection Criteria and Data Extraction

All human studies, observational (exploring habitual buckwheat intake), intervention studies/randomized controlled trials (RCT), and non-randomized trials (exploring buckwheat supplementation) or pre-clinical studies using human material were considered for inclusion. Further, animal, in vitro, or in vivo studies were eligible for inclusion. The studies were included if they (i) were conducted among individuals or animals of any age without GI conditions or with inflammatory bowel disease (IBD), irritable bowel syndrome (IBS), celiac disease (CeD), or those with GI tumors and (ii) investigated the association of buckwheat consumption with any of the following outcomes: (a) GI symptoms: bloating, abdominal pain, diarrhea, constipation, bowel inflammation, mucosal damage, (b) GI conditions: IBD, IBS, CeD or GI tumors focusing on the risk of developing a disease and changes in the course of the disease management and (c) GI microbiome: changes or differences in GI permeability, bacterial diversity, dysbiosis, microbiome metabolites and markers [SCFAs and trimethylamine oxide (TMAO)] (d) anti-inflammatory, anti-oxidant, or anti-tumor activity in the GI tract. The GI microbiome diversity and composition in this systematic review only included studies that employed sequencing techniques. Only studies that had documented their extraction or explicitly stated that the extracts were from buckwheat were included. Compounds commonly derived from buckwheat, such as rutin, quercetin, and D-fagomine, but that did not specify their source, were excluded. We also excluded letters to the editor, reviews, commentaries, and conference abstracts. 

The titles and abstracts were independently evaluated by two reviewers, and the full texts were assessed by two independent reviewers. In cases of disagreement, a consensus was sought by consulting a third reviewer. Two authors independently extracted the relevant information using a pre-defined data extraction form that included author and publication year, study design, population or sample characteristics, measurements or outcomes, and other relevant data. 

### 2.3. Methodological Quality Assessment

Two independent reviewers assessed the risks of bias in the studies. For the controlled one-arm non-randomized and observational trials, the National Heart Lung and Blood Institute Quality Assessment Tool [20,21] was used. For the animal in vivo and in vitro studies, the Toxicological data Reliability Assessment Tool (ToxRTool) [22] was used for assessment. The ToxRTool classifies the studies into three categories: reliable without restrictions, reliable with restrictions, or unreliable based on data reported by authors, including the quality and sources of their intervention, experimental design, and data analysis. This provided an assessment of the certainty of the findings and for characterizing the reliability of the results. 

## 3. Results

### 3.1. Literature Search and Study Characteristics

**Figure 1:**
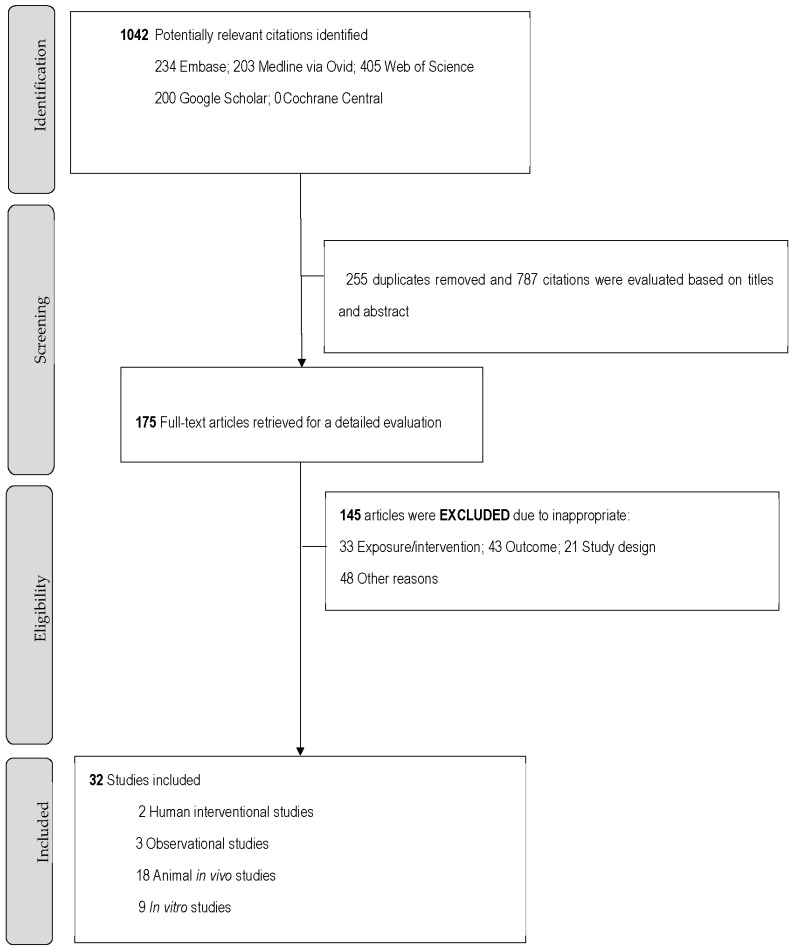
Flowchart of study inclusion in the review of the role of buckwheat consumption in GI health.

There were 1042 citations identified; after removing duplicates, 787 were included for the title and abstract screening, 175 full-text articles were retrieved, and 32 articles were included in the review (Figure 1). Among all included studies, 14 (44%) had human participants or human-derived samples (two interventional studies, three observational, and six in vitro studies) based on 1563 participants. The two human interventional studies included individuals with gluten sensitivity. One trial had individuals with non-celiac gluten sensitivity in a cross-over trial and the other compared children with celiac disease to those without the disease. The intervention included food products derived from buckwheat, such as flour, dough, pasta, and biscuits, and compared to a gluten-free diet, wheat, and placebo. The three observational studies focused on healthy adults and individuals with small intestine bacterial overgrowth (SIBO). The in vitro studies with GI-derived samples had fecal samples from healthy adults, human cancer cells, and ruminal fluid from cows. There were 19 (59%) animal studies included (18 in vivo studies and one in vitro study), which comprised 629 animals (mice, rats, piglets, lambs, and cows). Detailed characteristics of the included studies can be found in Table 1, Table 2, Table 3 and Table 4.

### 3.2. Buckwheat Consumption and the Development of GI Mucosal Inflammation and Symptoms

We identified three studies conducted among human participants or using human samples (one randomized controlled trial (RCT), one non-RCT, and one in vitro study); and six animal studies (four in mice, one in rats, and one in piglets), that investigated potential interactions between buckwheat and GI symptoms, Table 1 and Table 2. 

Two human trials and one observational study reported the role of buckwheat intervention/habitual intake on GI tolerance, Dinu et al. [23] randomly assigned adults diagnosed with non-CeD gluten sensitivity to either their standard gluten-free diet for 12 weeks or substituted their gluten-free diet with suitable commercially available buckwheat products. The trial consisted of a six-week intervention phase and a six-week control phase, then had a cross-over after each phase. At the end of the intervention period of this cross-over trial, adults consuming buckwheat products had significantly higher Symptom Severity Scale (SSS) scores vs. those on a gluten-free diet. During this period, the intervention group’s abdominal pain severity score and bloating severity were lower. There was no significant difference in non-CeD gluten sensitivity symptoms during the intervention period, but during the control period, there were significantly higher non-CeD gluten sensitivity symptoms such as nausea, joint/muscle pain, headache, attention disorder, and satisfaction with stool consistency in the majority of participants. The difference in the amount of food intake was not reported by the authors. In a non-randomized clinical trial, 30-day ingestion of buckwheat flour presented no toxicity to children with CeD, and no reactivity to buckwheat grain proteins was detected [24]. On the contrary, in the cross-sectional study of Zheng et al. [25], high daily consumption of buckwheat noodles was associated with a higher prevalence of IBS in Japanese adults regardless of sociodemographic, anthropometric, lifestyle profiles and other common Japanese food, i.e., rice, noodles, bread, and pasta. Meanwhile, in the studies of Pilipenko et al. [26,27], among individuals with a diagnosis of SIBO that were treated with anti-protozoan agents (tiliquinol and tilbroquinol), there were significantly higher resistance rates to treatment among those with higher consumption of buckwheat relative to those who consumed other cereals. 

Among in vitro/in vivo studies reporting on the effect of buckwheat on GI inflammation, one study used human and six used animal material. In an in vitro study on a human myofibroblast colon CCD-18Co cell line, intestinal inflammation was tested by applying flavonoids extracted from buckwheat groats and sprouts and showed significantly lower levels of tumor necrosis factor-alpha (TNFα) induced colonic myofibroblast migration [28]. This indicates that buckwheat bioactive compounds are linked to lower intestinal inflammation. Six in vivo animal studies explored the potential of buckwheat to improve GI mucosa lesions and inflammation. Two in vivo studies in mice explored if the anti-inflammatory activity of buckwheat sprout extract and pollen derived from buckwheat improved the intestinal barrier among ceftriaxone-treated mice [29,33]. Mice that received lipopolysaccharide (LPS) and buckwheat sprout extract had lower interleukin 6 (IL 6) and TNFα in their spleen and liver and had lower serum cytokines compared with mice given oral LPS alone [29]. On the other hand, mice exposed to buckwheat pollen were able to restore their intestinal barrier and had higher secretion of soluble intestinal IgA and lower GI inflammation compared to those that had natural resolution [33]. Buckwheat bran, likewise, significantly improved gastric mucosal lesions in indomethacin-induced lesions compared to other food tested (wheat, rice, soybean, peanuts, and maize) [30]. Similarly, feeding mice with buckwheat alleviated gastritis in mice that received a high salt diet (an animal model in studying gastric inflammation), and the lymphocyte infiltration in the submucosa among buckwheat-fed mice was lower or absent compared to those who had a high salt diet without buckwheat. Pro-inflammatory factors in the stomach tissue of buckwheat-fed mice were similar to mice that received a low salt diet [32]. When comparing rats on a high-fat diet to those fed with a normal diet, a high-fat diet with fermented milk and a high-fat diet with buckwheat for four weeks led to lower pro-inflammatory markers in the colon [34]. Finally, in the study that included piglets [31], buckwheat, in combination with probiotics, was unable to influence the intestinal histomorphology and induction of the mucosal immune system among piglets. 

### 3.3. Buckwheat and Its GI Anti-Cancer Cell Line Activity

Seven in vitro studies used human GI-derived colon and gastro-hepatic cancer cell lines, and one animal study focused on the potential of buckwheat-derived compounds as an agent against tumor growths (Table 3). Dziedzic et al. [35] studied the inhibitory effect of buckwheat grains and groats after in vitro digestion on human colon adenocarcinoma cell line HT-29. They found that buckwheat bran has the highest cytotoxicity to the cancer cell line, but this activity was lower in raw and roasted grains. The same study determined that flavonoids catechin and quercetin, as well as the amino acids serine, proline, glycine, histidine, and arginine as the most cytotoxic for the cancer cells. While rutin was the most abundant flavonoid measured, it did not show significant cytotoxicity. The studies of Ishii et al. [29], and Swiatecka et al. [36], used colon cancer lines to test buckwheat-derived products but showed conflicting results. Ishii et al. [29] compared the LPS-induced response from human carcinoma cell lines (CoLoTC) and extracts from buckwheat. They used IL8 as a biomarker for inflammation and found that the buckwheat extracts significantly lowered IL8 expression in colon cancer cells in a dose-dependent manner but did not influence cell viability. On the other hand, Swiatecka et al. [36] tested buckwheat protein hydrolysate on the CaCo-2 cell line, resulting in significantly lower cell proliferation and significantly higher levels of IL8. In another study, Kim et al. [37] prepared extracts from buckwheat hull with multiple fractions of different solvents (ethanol, ethyl acetate, hexane, butanol, chloroform, and water) and tested them to multiple cancer lines (human gastric carcinoma, human hepatocellular, carcinoma, human lung carcinoma, human breast adenocarcinoma, and human cervical adenocarcinoma). Against human gastric carcinoma, all fractions of buckwheat hull extract, except water, showed above 80% inhibition, while only hexane and butanol fractions inhibited hepatocellular carcinoma at the same level. A similar situation was seen in the experiments of Zhou et al. [38,39], wherein they tested flavonoid extracts from Tartary buckwheat and exposed the MGC80-3 human gastric cancer cell line to them. The complex flavonoid extracts were able to inhibit gastric cancer cell proliferation. In the study by Li et al. [40], flavonoid extracts from buckwheat were tested against human hepatoma HepG2 cells. They found that the flavonoids were potent anti-oxidants, with quercetin as the most potent, and the flavonoids were able to inhibit HepG2 cell proliferation in a dose and time-dependent manner. These in vitro studies support the animal in vivo observations of Liu et al. [41], wherein they induced colon tumors in male rats with 1,2-dimethylhydrazine (DMH) and then examined the effect of buckwheat protein consumption in comparison to casein on these tumors. Even though there was no significant difference in colonic tumor incidence and size, the development of colon adenocarcinomas was 47% lower among those fed with buckwheat. Likewise, there was significantly lower colonic epithelium proliferation and lower bloody stool incidence among those fed with buckwheat compared to those given casein.

### 3.4. Effects of Buckwheat on the GI Microbiome

Sixteen (one human in vitro, one animal in vitro, and 14 animals in vivo) studies looked into the effects of buckwheat on the GI microbiome (Table 4). Among these studies, 13 used genomic sequencing techniques in identifying and quantifying the taxonomic classification of the microorganisms, and 11 studies reported the measurement of fecal SCFAs. Of 14 animal in vivo studies, seven studied mice models, five were on rats, one with lambs, and one in piglets. 

The study of Jiang et al. [42] studied phenolics and carbohydrates of common buckwheat honey and their effects on the human intestinal microbiome using fecal samples from healthy adults. They had eight experimental groups, a positive control with fructo-oligosaccharide, a negative control, and the original fecal sample. Buckwheat honey groups and the positive control exhibited significantly lower Chao and Shannon indexes and higher Simpson indexes compared to the negative control. These indices are measures of diversity that look at the sample’s total number of organisms or how these organisms are represented in the sample. There was likewise clustering of the experimental groups with the positive control and a distinct separation from the negative control in the ordination visualization, indicating a difference in beta diversity. 

Among animal studies, six studies investigated common buckwheat and reported differences in the fecal microbiome in comparison to a high-salt diet, high-fat diet, or normal diet. In Supplemental Figure S1, we summarized the findings on the differences in GI microbiota from animal studies using common buckwheat as an intervention. Huang et al. [47] studied groups of mice with a normal diet, a high-fat diet, a high-fat diet with simvastatin, a high-fat diet with a low dose of common buckwheat dried powder and a high-fat diet with a high dose of buckwheat for eight weeks. High-fat diet had a significantly different fecal microbiome compared to a normal diet. Among diet groups with high fat, high-dose supplementation of buckwheat was significantly different from the solely high-fat diet. Likewise, a high-fat diet significantly lowers fecal SCFAs vs. normal diet, but supplementation of simvastatin or with a high dose of buckwheat had higher fecal levels of total SCFAs, acetic acid, and butyric acid compared to a high-fat diet [47]. Li et al. [32] explored differences in the fecal microbiome of mice with high-salt diet-induced gastritis and found that the high-salt diet groups had significantly different alpha and beta diversity compared to a normal diet. Thereafter, supplementation with buckwheat for four weeks was conducted, and they observed that the fecal microbiome of those with buckwheat was more similar to a normal diet compared to a high salt diet. In the study of Zhu et al. [33], they compared a group of mice who had no antibiotic exposure, a ceftriaxone group, post-ceftriaxone exposure with a natural resolution, and post-ceftriaxone exposure groups with varying amounts of common buckwheat pollen extracts for three weeks. The group with buckwheat had significantly higher Shannon, Chao, and Ace indices compared to those who had not been given any buckwheat. Moreover, the group exposed to buckwheat pollen extracts had a higher abundance of sIgA secretion-related bacteria and inflammation-related bacteria [33]. On the other hand, an increasing amount of common buckwheat straw in the diet of the lambs led to lower alpha diversity in the gut with a linear decrease in Firmicutes and an increase in Bacteroidetes. This is coupled with significantly higher total volatile fatty acids, propionate, and butyrate but lower acetate [52]. In rats receiving common buckwheat flour, they had significantly higher concentrations of acetate, butyrate, and total SCFAs compared to casein and the soya protein isolate group [51]. In a study that explored the use of common buckwheat as feeds in cows, they reported no significant difference in the ruminal production of SCFAs vs. grass clover hay or in increasing common buckwheat concentration compared to the basal diet [43].

Nine studies applied Tartary buckwheat and reported differences in the animal fecal microbiome in comparison to a high-fat diet [7,34,44,45,46,48,49,50] and basal diet [53]. In Supplemental Figure S2, we summarized the findings on differences in GI microbiota from animal studies using Tartary buckwheat as an intervention. Zhou et al. [44] reported significantly higher *Bifidobacterium, Lactobacillus, Enterococcus*, and *Clostridium* and a significant decrease in *Escherichia* and *Bacteroides* in mice who received a high-fat diet with Tartary buckwheat-resistant starch compared to that of the high-fat diet with casein. This was in line with another publication from the same leading author [46]; they reported significantly higher acetic acid, propionic acid, and butyric acid with buckwheat vs. casein supplementation in high-fat diets among mice. In another study by Zhou et al. [45], the alpha diversity was not significantly different in Shannon, Ace, and Chao 1 indices among the groups. The microbial composition of the high-fat diet with Tartary buckwheat-resistant starch was closest to the bacterial phylum and genus profile of the low-fat diet. The high-fat diet groups had higher acetate compared to the low-fat diet, but the presence of Tartary buckwheat-resistant starch had significantly higher concentrations of propionate, butyrate, and total SCFAs when compared to the high-fat diet group. Liu et al. [7] studied two groups of rats that were initially given a normal diet and a high-fat diet for six weeks. The high-fat diet group was then divided into three groups and given no treatment, simvastatin and Tartary buckwheat protein for five weeks. The rats on the high-fat diet had significantly lower alpha diversity (Shannon and Simpson indices) compared to controls, but buckwheat protein supplementation improved the alpha diversity when compared to the groups with a high-fat diet. Likewise, the group with Tartary buckwheat protein supplementation had a modestly different GI microbiome vs. rats with a high-fat diet. The high-fat diet lowered fecal SCFAs vs. the normal diet, but those with buckwheat protein supplementation had significantly higher total SCFAs, acetic acid, and propionic acid compared to rats with a high-fat diet only. In another study that compared three types of intervention in rats (normal diet, high-fat diet with fermented milk, and high-fat diet with Tartary buckwheat fermented milk), there was no significant difference in the Shannon and Simpson indices in the groups, but there was significantly higher Chao and Ace indices in the group with Tartary buckwheat fermented milk compared to those with fermented milk only and the normal diet groups. The fecal SCFAs of the two groups with high-fat diets were significantly higher than the control group, and the group with Tartary buckwheat fermented milk had higher SCFAs but were not statistically different from the group with fermented milk [34]. Ren et al. [49] compared six interventions in rats; the high-fat diet group had significantly lower Shannon and Simpson diversity indices vs. normal diet but were significantly higher vs. groups with Tartary buckwheat. Those with black Tartary buckwheat seeds and germinated black Tartary buckwheat had significantly different microbiome compositions among those groups fed with a high-fat diet. Fecal SCFAs were significantly higher in a high-fat diet vs. a normal diet, and Tartary buckwheat supplementation (seeds or germinated) did not increase the SCFAs concentration. Similarly, in another study among rats, a high-fat diet decreased the alpha diversity measures compared to a normal diet, but the Tartary buckwheat powder supplementation group had significantly higher Chao index and were not significantly different from the Shannon and Simpson indices and observed species vs. high-fat diet [50]. Tartary buckwheat powder supplementation had no effect on the fecal SCFAs concentration. Wu et al. [48] studied the antidiabetic effect of the soluble dietary fibers from Tartary buckwheat brans using mice with induced diabetes by feeding with a high-fat diet for five weeks. The diabetic groups were then given a varying (low, moderate, and high) dose of buckwheat fibers for eight weeks. They found that acetate and propionate were significantly higher with Tartary buckwheat bran supplementation vs. the healthy and diabetic controls. Butyrate, however, was only significantly higher in moderate and high doses compared to healthy and diabetic controls.

### 3.5. Study Quality

Among the five human interventional and observational studies, four had a moderate risk of bias, and one had a high risk of bias. The majority (19/27) of the in vitro and animal in vivo studies were reliable without restrictions, and six studies were reliable with restrictions. The studies with restrictions generally suffer from the lack of description of the properties of the buckwheat they were testing. Some of the studies used crude extracts and in vitro digestates but did not provide information on the properties of the test substance. Detailed descriptions of the risk of biases are found in Supplemental Tables S1–S3.

## 4. Discussion

The current evidence suggests that buckwheat may have some anti-inflammatory properties that could potentially lower GI mucosal lesions and lower GI mucosal inflammation among individuals with gluten sensitivity. In vitro studies show cytotoxic properties against human GI cancer cell lines. Likewise, animal studies showed that groups with buckwheat consumption had GI microbiome alpha diversity measures similar to those with a normal diet and could have higher levels of fecal SCFAs. The findings, however, are based only on a limited number of studies involving humans and are mostly based on animal models. 

Buckwheat has an abundance of bioactive compounds, such as flavonoids and steroids that have been shown to be anti-inflammatory [5,54,55,56,57]. Proposed mechanistic actions involve the NF-kB and mitogen-activated protein kinase (MAPK) pathways leading to inhibition of inflammatory mediators such as nitric oxide, IL6, TNFa, and reduced expression of inducible nitric oxide synthase (iNOS) and cyclooxygenase-2 (COX-2) [55,57,58,59]. Likewise, the high abundance of polyphenols, flavonoids, polysaccharides, and proteins have been shown to be responsible for buckwheat’s cytotoxic activity against cancer cells wherein they induce cell apoptosis, increase expression of apoptotic proteins such as caspase-8, promote cell cycle arrest, and increase reactive oxygen species (ROS) [5,39,40,60]. Moreover, the potency of the buckwheat bioactive compounds is heightened by the low absorption in the GI tract of some of these compounds, thereby leading to longer exposure in the GI mucosa and thus the observed beneficial effects seen with buckwheat consumption [61,62,63]. These potent anti-inflammatory properties of buckwheat make it a good candidate as a food source for individuals with inflammatory bowel diseases. 

Buckwheat consumption or supplementation affected the GI microbiome as well and has been shown to either have higher or lower alpha diversity. Alpha diversity is a measure of the microorganism’s richness and evenness within samples [64]. A high-fat diet in the studies has significantly lower alpha diversity compared to healthy controls, and those who consume buckwheat have higher alpha diversity. High alpha diversity is not necessarily beneficial as this could signify a disturbance in the microbiome and an abundance of rare microbial taxonomic groups. Rare and opportunistic organisms tend to proliferate when the core microbiome is disturbed, and there is an inability to control the growth of new organisms. Therefore, comparing the beta diversity to a healthy control provides information on whether the observed changes in the alpha diversity are beneficial. Beta diversity is the difference between identified taxonomic groups in different sites or conditions [65]. In this review, the GI microbiome composition of animals that consumed buckwheat or those supplemented with buckwheat clusters approaches healthy control GI microbiome profiles. The studies, however, have not statistically measured the beta diversity and relied only on visualization through ordination techniques. 

Moreover, the bacterial phylum such as Firmicutes and Bacteroidetes, family Akkermansiaceae and its genus *Akkermansia* and the family Lachnospiraceae in the animal studies have been shown to significantly differ in their relative abundance compared to their baseline after buckwheat consumption but are inconsistent in the direction of difference across the studies. This could be due to the heterogeneity of animal models used, study design differences, especially the length of exposure to buckwheat, different formulations and species of buckwheat used, and comparisons relying on relative abundance with compositional data. The microbiome data in the studies are compositional except for those studies that employed targeted sequencing wherein they quantified the absolute abundance of specific bacteria. Directly comparing the relative abundance in compositional data is problematic as the relative abundance of a group of bacteria could be affected by the increase or decrease of other bacteria in the sample and not inherently to the bacteria in question. Therefore, the changes could be artificial. The two studies that quantified their targeted bacteria showed increases in the absolute abundance of beneficial bacteria such as *Bifidobacterium*, *Lactobacillus*, and *Enterococcus*. These groups of bacteria are known to be fermenters of soluble dietary fibers that produce SCFAs [66]. Animal groups with buckwheat consumption had significantly higher fecal SCFAs, as seen in this review. These short-chain fatty acids are beneficial in the gut as they enhance mucin production for GI mucosa protection, increase expression of GI tight junctions, limit GI inflammation, serve as an energy source for colonic cells and increase the colonic pH, thus preventing the growth of harmful bacteria [67,68].

The evidence of the higher prevalence of IBS and SIBO observed in those that consume moderate to high amounts of buckwheat could be that individuals with IBS and SIBO are more likely to integrate the buckwheat into their regular diet, considering it is gluten-free and low in FODMAP. However, considering the cross-sectional nature of the evidence, this remains unclear. There are reports of a significant incidence of allergic reactions to buckwheat consumption, which presents with a range of symptoms from urticaria to more serious conditions such as anaphylaxis [5,69,70]. These individuals could represent individuals with minor and non-systematic reactions to buckwheat. Subtle allergic reactions could precipitate inflammatory reactions in the gut mucosa and cause improper digestion, leading to abdominal pain, bloating, increased GI transit, and other GI symptoms that are related to IBS and SIBO. 

### Strengths and Limitations

This review was prepared using published guidelines and the best available tools to assess the risk of bias. To our knowledge, this is the first report that systematically reviewed available literature on GI symptoms, cytotoxicity towards GI-derived cancer cells, and GI microbiome changes after buckwheat consumption. We did a sensitive search strategy to reduce the risk of publication bias and in order to identify as many relevant studies as possible. However, we were not able to search all existing online databases. We had no restrictions on language or year of publication, but we may have missed articles published in other languages other than English. The included studies were of different study designs and of diverse populations, and thus the substantial heterogeneity did not allow pooling or results. For studies based on animal models, we defined whether a positive, negative, or no effect was reported; otherwise, we reported the magnitude of the effect/association, direction, and significance.

We acknowledge that our findings were based predominantly on animal models. The GI dynamics in animals may not be similar to the processes and microbial composition of the human GI tract, but they do provide good insights into possible research areas to explore. The anti-inflammatory, cytotoxic capacity, and microbiome changes in buckwheat consumption could benefit from human clinical trials. Moreover, common buckwheat and Tartary buckwheat are separate species of plants, and the abundance of their bioactive compounds differ and may result in different effects as well. Likewise, there are reports on buckwheat allergic reactions not related to gluten sensitivity. The design of future clinical trials should put this into consideration. Improvement of tools and analytical methods in microbiome studies allows for better analysis in handling high throughput compositional data. Therefore, the analytical methods used should be robust enough for this data type to avoid spurious correlations and better interpretation of the data gathered. 

## 5. Conclusions

Current evidence, based mainly on data from animal models, suggests buckwheat may have some potential health benefit in the GI tract mediated via its anti-inflammatory, antioxidant, and cancer cell line inhibiting potential. In addition, changes in microbiome and SCFAs are observed due to buckwheat intake, but their impact on health in humans remains to be elucidated.

## Figures and Tables

**Table 1:** Summarized findings on the role of buckwheat consumption on GI mucosal inflammation and symptoms in humans.

Lead Author, Year; Country	Study Type	Population Characteristic	Sample Size; Follow-Up (mos)	GI Outcome Finding	Risk of Bias
Dinu, 2017; Italy	RCT Crossover	Adults with non-CeD gluten sensitivity	19 3 (2 periods)	↓ abdominal pain and bloating vs. gluten-free diet	Moderate [23]
De Francischi, 1994; Brazil	non-RCT	Children with CeD	4 1	⇄ toxic prolamines to children CeD	High [24]
Zheng, 2015; Japan	Observational	Healthy adults and adults with IBS	1082 36	↑ prevalence of IBS in moderate to high buckwheat consumption regardless of socio-demographic profile, anthropometric, and lifestyle-related factors	Moderate [25]
Pilipenko, 2019; Pilipenko, 2018; Russia	Observational	Individuals with SIBO	458 2	↑ resistance to SIBO (0.41 ± 0.47 high buckwheat consumption vs. 0.14 ± 0.35 relative to the rate of consumption of cereals, *p* < 0.001) compared to those with a resolution of SIBO	Moderate [26,27]
Giménez-Bastida, 2018; USA	In vitro with human myofibroblasts of colon CCD-18Co cell line	-	-	↓ TNFα induced colonic myofibroblast migration vs. control	Reliable w/o restrictions [28]

RCT, randomized controlled trial; CeD, celiac disease; SIBO, small intestine bacterial overgrowth. ↑ higher, ↓ lower, ⇄ no difference - no data.

**Table 2:** Summarized findings on the role of buckwheat consumption on GI mucosal inflammation and symptoms in animals.

Lead Author, Year; Country	Study Type	Population Characteristic	Sample Size;Follow-Up (mos)	GI Outcome Finding	Risk of Bias
Ishii, 2008; Japan	Animal In vivo	Mice	10 2	↓ IL6 and TNFα in the spleen and liver vs. oral LPS	Reliable w/o restrictions [29]
Afroz 2016; Japan	Animal In vivo	Mice	35 1.25	↓ gastric mucosal lesions	Reliable with restrictions [30]
Gāliņa, 2020; Latvia	Animal In vivo	Pig	44 1.5	⇄ histomorphology and immune system of the intestinal mucosa	Reliable w/o restrictions [31]
Li, 2020; China	Animal In vivo	Mice	40 2	↓ gastric mucosa inflammation vs. high salt diet ↓ lymphocyte infiltration vs. high salt diet	Reliable w/o restrictions [32]
Zhu, 2020; China	Animal In vivo	Mice	60 2	↓ intestinal mucosal and recess destruction vs. natural resolution ↓ intestinal inflammation vs. natural resolution	Reliable w/o restrictions [33]
Zhou, 2019, China	Animal In vivo	Rats	32 1	↓ colon IL6, TNFa, and LPS with a high-fat diet with buckwheat vs. a high-fat diet	Reliable w/o Restrictions [34]

IL6, interleukin 6; TNFa, tumor necrosis factor alpha; LPS, lipopolysaccharides. ↑ higher, ↓ lower, ⇄ no difference - no data.

**Table 3:** Summarized findings on the role of buckwheat consumption on GI-derived cancer cells and tumors.

Lead Author, Year; Country	Sample Source; Study Type	Buckwheat Preparation; Control	GI Derived Cancer Cell Line Tested	Significant Finding	Risk of Bias
Dziedzic, 2018; Poland	Human In vitro	Buckwheat digestate;Blank cell culture	Human colon adenocarcinoma cell line HT-29	+ cytotoxicity capacity of buckwheat bran, groats, and raw grain	Reliable with restrictions [35]
Ishii, 2008; Japan	Human In vitro	Buckwheat ethanol extract; LPS	Human colon cancer cell line (CoLotC)	↓ IL8 expression ⇄ cytotoxicity	Reliable w/o restrictions [29]
Swiatecka, 2013; Poland	Human In vitro	Buckwheat protein hydrolysate; Blank cell culture	CaCo-2 cell line	+ cytotoxicity ↑ IL8 expression	Reliable with restrictions [36]
Kim, 2007; Korea	Human In vitro	Buckwheat ethanol extract None	Gastric carcinoma cell line, Hepatocellular carcinoma	+ cytotoxicity in a dose-response manner	Reliable with restrictions [37]
Zhou, 2013; China	Human In vitro	Buckwheat flavonoids extract; Blank cell culture	Human gastric cancer MGC80-3	+ cytotoxicity	Reliable w/o restrictions [38]
Zhou, 2019; China	Human In vitro	Buckwheat flavonoids extract; Blank cell culture	Human gastric cancer MGC80-3	+ cytotoxicity	Reliable w/o restrictions [39]
Li, 2014; China	Human In vitro	Buckwheat flavonoids extract; Blank cell culture	Human hepatoma HepG2 cells	+ cytotoxicity in a dose-response and time-dependent manner + anti-oxidant capacity	Reliable w/o restrictions [40]
Liu, 2001; Japan	Rat In vivo	Buckwheat proteins Casein	1,2-dimethyl hydrazine-induced colonic tumors	↓ incidence of bloody stools ⇄ incidence of colonic tumors ↓ proliferation of colonic epithelium	Reliable with restrictions [41]

IL8, interleukin 8; LPS, lipopolysaccharides. ↑ higher, ↓ lower, ⇄ no difference, + present.

**Table 4:** Summarized findings on the role of buckwheat consumption on GI microbiome.

Lead Author, Year; Country	Population Study Type Type of Buckwheat Diet Comparison	Microbial Diversity Findings		SCFAs				Risk of Bias
		α Diversity	β Diversity	Acetate	Propionate	Butyrate	Total	
Jiang, 2020; China	Healthy adult In vitro Common buckwheat Negative control	↓ Chao1 and Shannon indices ↑ Simpson index	+ β diversity among buckwheat groups vs. negative control	-	-	-	-	Reliable w/o restrictions [42]
Amelchanka, 2010; Switzerland	Cows In vitro Common buckwheat Basal diet/grass clover hay	-	-	⇄	⇄	⇄	⇄	Reliable w/ restrictions [43]
Li, 2020; China	Mice In vivo Common buckwheat High salt diet	↑ α diversity	Influenced the return to control microbiome profile after a high salt diet	-	-	-	-	Reliable w/o restrictions [32]
Zhou, 2018; China	Mice In vivo Tartary buckwheat High-fat diet	-	-	↑	↑	↑	-	Reliable w/o restrictions [44]
Zhou, 2019; China	Mice In vivo Tartary buckwheat High-fat diet	-	-	↑	↑	↑	-	Reliable w/o restrictions [45]
Zhou, 2020; China	Mice In vivo Tartary buckwheat High-fat diet	⇄ Shannon, Chao, and Ace indices	-	⇄	↑	↑	↑	Reliable w/o restrictions [46]
Huang, 2020; China	Mice In vivo Common buckwheat High-fat diet	-	High-dose buckwheat consumption has significantly different β-diversity	↑	⇄	↑	↑	Reliable w/o restrictions [47]
Wu, 2021; China	Mice In vivo Tartary buckwheat None	-	-	↑	↑	↑	-	Reliable w/o restrictions [48]
Zhu, 2020; China	Mice In vivo Common buckwheat Natural resolution after ceftriaxone exposure	↑ Shannon, Chao, and Ace indices	-	-	-	-	-	Reliable w/o restrictions [33]
Liu, 2021; China	Rats In vivo Tartary buckwheat High-fat diet	↑ α diversity	-	↑	↑	⇄	↑	Reliable w/o restrictions [7]
Zhou, 2019; China	Rats In vivo Tartary buckwheat High-fat diet	⇄ Shannon and Simpson indices ↑ Chao and Ace indices	-	⇄	⇄	⇄	⇄	Reliable w/o restrictions [34]
Ren, 2021; China	Rats In vivo Tartary buckwheat High-fat diet	↑ Shannon and Simpson indices	BTB and GTB have significantly different β-diversity with a high-fat diet	-	-	-	-	Reliable w/o restrictions [49]
Peng, 2019; China	Rats In vivo Tartary buckwheat High-fat diet	↑ Chao index	-	⇄	⇄	⇄	-	Reliable w/o restrictions [50]
Fotschki, 2020; Poland	Rats In vivo Common buckwheat Normal diet	-	-	↑	⇄	↑	↑	Reliable w/o restrictions [51]
Mu, 2019; China	Lambs In vivo Common buckwheat Normal diet	↓ Chao1, Ace, Shannon, and Simpson indices with increasing buckwheat concentration	-	↓	↑	↑	↑	Reliable w/o restrictions [52]
Cui, 2019; China	Pig In vivo Tartary buckwheat Basal diet	⇄ Observed species and Chao, Shannon, and Simpson indices	-	-	-	-	-	Reliable w/o restrictions [53]

SCFA, short chain fatty acid; BTB, black Tartary buckwheat; GTB, germinating Tartary buckwheat; ↑higher, ↓ lower, ⇄ no difference, - no data.

## Data Availability

Not applicable.

## Supplementary Materials

The following supporting information can be downloaded at: https://www.mdpi.com/article/10.3390/nu15010001/s1, Supplemental Appendix SI. PRISMA checklist for the systematic review of human and animal evidence on the role of buckwheat consumption on gastrointestinal health [71]. Supplemental Appendix SII. The study protocol of the systematic review of human and animal evidence on the role of buckwheat consumption on gastrointestinal health; Supplementary Table S1. Quality assessment of controlled intervention studies in the review of the role of buckwheat consumption on GI health using the National Heart Lung and Blood Institute Quality Assessment Tool; Supplementary Table S2. Quality assessment of observational studies in the review of the role of buckwheat consumption on GI health using the National Heart Lung and Blood Institute Quality Assessment Tool; Supplementary Table S3. Quality assessment of in vitro and animal in vivo studies in the review of the role of buckwheat consumption on GI health using the ToxRTool; Supplemental Figure S1. Changes in communities comparing intervention with common buckwheat and control; Supplemental Figure S2. Changes in communities comparing intervention with Tartary buckwheat and control.

## References

[1] Ji X., Han L., Liu F., Yin S., Peng Q., Wang M. (2019). A mini-review of isolation, chemical properties and bioactivities of polysaccharides from buckwheat (*Fagopyrum* Mill). Int. J. Biol. Macromol..

[2] Kreft M. (2016). Buckwheat phenolic metabolites in health and disease. Nutr. Res. Rev..

[3] Raguindin P.F., Itodo O.A., Stoyanov J., Dejanovic G.M., Gamba M., Asllanaj E., Minder B., Bussler W., Metzger B., Muka T. (2021). A systematic review of phytochemicals in oat and buckwheat. Food Chem..

[4] Zhu F. (2020). Dietary fiber polysaccharides of amaranth, buckwheat and quinoa grains: A review of chemical structure, biological functions and food uses. Carbohydr. Polym..

[5] Zou L., Wu D., Ren G., Hu Y., Peng L., Zhao J., Garcia-Perez P., Carpena M., Prieto M.A., Cao H. (2021). Bioactive compounds, health benefits, and industrial applications of Tartary buckwheat (*Fagopyrum tataricum*). Crit. Rev. Food Sci. Nutr..

[6] Noreen S., Rizwan B., Khan M., Farooq S. (2021). Health Benefits of Buckwheat (*Fagopyrum Esculentum*), Potential Remedy for Diseases, Rare to Cancer: A Mini Review. Infect. Disord. Drug Targets.

[7] Liu J., Song Y., Zhao Q., Wang Y., Li C., Zou L., Hu Y. (2021). Effects of Tartary Buckwheat Protein on Gut Microbiome and Plasma Metabolite in Rats with High-Fat Diet. Foods.

[8] Ajamian M., Rosella G., Newnham E.D., Biesiekierski J.R., Muir J.G., Gibson P.R. (2021). Effect of Gluten Ingestion and FODMAP Restriction on Intestinal Epithelial Integrity in Patients with Irritable Bowel Syndrome and Self-Reported Non-Coeliac Gluten Sensitivity. Mol. Nutr. Food Res..

[9] Ruan W., Engevik M.A., Spinler J.K., Versalovic J. (2020). Healthy Human Gastrointestinal Microbiome: Composition and Function After a Decade of Exploration. Dig. Dis. Sci..

[10] Heiman M.L., Greenway F.L. (2016). A healthy gastrointestinal microbiome is dependent on dietary diversity. Mol. Metab..

[11] Valido E., Stoyanov J., Bertolo A., Hertig-Godeschalk A., Zeh R.M., Flueck J.L., Minder B., Stojic S., Metzger B., Bussler W. (2021). Systematic Review of the Effects of Oat Intake on Gastrointestinal Health. J. Nutr..

[12] Li L., Lietz G., Seal C. (2018). Buckwheat and CVD Risk Markers: A Systematic Review and Meta-Analysis. Nutrients.

[13] Norbäck D., Wieslander G. (2021). A Review on Epidemiological and Clinical Studies on Buckwheat Allergy. Plants.

[14] Huda M.N., Lu S., Jahan T., Ding M., Jha R., Zhang K., Zhang W., Georgiev M.I., Park S.U., Zhou M. (2021). Treasure from garden: Bioactive compounds of buckwheat. Food Chem..

[15] Muka T., Glisic M., Milic J., Verhoog S., Bohlius J., Bramer W., Chowdhury R., Franco O.H. (2020). A 24-step guide on how to design, conduct, and successfully publish a systematic review and meta-analysis in medical research. Eur. J. Epidemiol..

[16] Moher D., Liberati A., Tetzlaff J., Altman D.G., The PRISMA Group (2009). Preferred Reporting Items for Systematic Reviews and Meta-Analyses: The PRISMA Statement. J. Clin. Epidemiol..

[17] Rethlefsen M.L., Kirtley S., Waffenschmidt S., Ayala A.P., Moher D., Page M.J., Koffel J.B., PRISMA-S Group (2021). PRISMA-S: An extension to the PRISMA Statement for Reporting Literature Searches in Systematic Reviews. Syst. Rev..

[18] Harzing A.W. (2007). Publish or Perish. https://harzing.com/resources/publish-or-perish.

[19] Bramer W.M., Giustini D., de Jonge G.B., Holland L., Bekhuis T. (2016). De-duplication of database search results for systematic reviews in EndNote. J. Med. Libr. Assoc..

[20] NIH National Heart Lung and Blood Institute Quality Assessment Tool for before-after (Pre-Post) Studies with no Control Group. https://www.nhlbi.nih.gov/health-topics/study-quality-assessment-tools.

[21] NIH National Heart Lung and Blood Institute Quality Assessment of Controlled Intervention Studies. https://www.nhlbi.nih.gov/health-topics/study-quality-assessment-tools.

[22] Schneider K., Schwarz M., Burkholder I., Kopp-Schneider A., Edler L., Kinsner-Ovaskainen A., Hartung T., Hoffmann S. (2009). “ToxRTool”, a new tool to assess the reliability of toxicological data. Toxicol. Lett..

[23] Dinu M., Macchia D., Pagliai G., Gori A.M., Cesari F., Marcucci R., Sofi F., Casini A. (2017). Symptomatic efficacy of buckwheat products in Non-Celiac Gluten Sensitivity (NCGS). Asia Pac. J. Clin. Nutr..

[24] De Francischi M.L.P., Salgado J., Da Costa C.P. (1994). Immunological analysis of serum for buckwheat fed celiac patients. Mater. Veg..

[25] Zheng Z., Huang C., Guo Y., Niu K., Momma H., Kobayashi Y., Fukudo S., Nagatomi R. (2015). Staple Foods Consumption and Irritable Bowel Syndrome in Japanese Adults: A Cross-Sectional Study. PLoS ONE.

[26] Pilipenko V.I., Isakov V.A., Morozov S.V., Vlasova A.V., Naydenova M.A. (2019). Association of food patterns with different forms of small intestinal bacterial overgroth syndrome and treatment efficacy. Ter. arkhiv.

[27] Pilipenko V., Isakov V.A., Vlasova A.V., Naidenova M.A. (2019). Features of nutrition pattern of patients with small intestinal bacterial overgrowth resistant to therapy. Vopr Pitan.

[28] Giménez-Bastida J.A., Laparra-Llopis J.M., Baczek N., Zielinski H. (2018). Buckwheat and buckwheat enriched products exert an anti-inflammatory effect on the myofibroblasts of colon CCD-18Co. Food Funct..

[29] Ishii S., Katsumura T., Shiozuka C., Ooyauchi K., Kawasaki K., Takigawa S., Fukushima T., Tokuji Y., Kinoshita M., Ohnishi M. (2008). Anti-Inflammatory Effect of Buckwheat Sprouts in Lipopolysaccharide-Activated Human Colon Cancer Cells and Mice. Biosci. Biotechnol. Biochem..

[30] Afroz S., Ikoma T., Yagi A., Kogure K., Tokumura A., Tanaka T. (2016). Concentrated Phosphatidic Acid in Cereal Brans as Potential Protective Agents against Indomethacin-Induced Stomach Ulcer. J. Agric. Food Chem..

[31] Gāliņa D., Ansonska L., Valdovska A. (2020). Effect of Probiotics and Herbal Products on Intestinal Histomorphological and Immunological Development in Piglets. Veter Med. Int..

[32] Li Y., Li W., Wang X., Ding C., Liu J., Li W., Sun Y. (2020). High-Salt Diet-Induced Gastritis in C57BL/6 Mice is Associated with Microbial Dysbiosis and Alleviated by a Buckwheat Diet. Mol. Nutr. Food Res..

[33] Zhu L., Li J., Wei C., Luo T., Deng Z., Fan Y., Zheng L. (2020). A polysaccharide from *Fagopyrum esculentum* Moench bee pollen alleviates microbiota dysbiosis to improve intestinal barrier function in antibiotic-treated mice. Food Funct..

[34] Zhou Y., Jiang Q., Zhao S., Yan B., Zhou X. (2019). Impact of Buckwheat Fermented Milk Combined with High-Fat Diet on Rats’ Gut Microbiota and Short-Chain Fatty Acids. J. Food Sci..

[35] Dziedzic K., Górecka D., Szwengiel A., Olejnik A., Rychlik J., Kreft I., Drożdżyńska A., Walkowiak J. (2018). The cytotoxic effect of artificially digested buckwheat products on HT-29 colon cancer cells. J. Cereal Sci..

[36] Świątecka D., Markiewicz L.H., Wroblewska B. (2013). In vitro evaluation of the effect of the buckwheat protein hydrolysate on bacterial adhesion, physiology and cytokine secretion of Caco-2 cells. Central Eur. J. Immunol..

[37] Kim S.-H., Cui C.-B., Kang I.-J., Kim S.Y., Ham S.-S. (2007). Cytotoxic Effect of Buckwheat (*Fagopyrum esculentum* Moench) Hull Against Cancer Cells. J. Med. Food.

[38] Zhou X.L., Meng X.X., Wang Q., Zhou Y.M., Li Z.J. (2013). Comparative Anti-Tumor Activity Study of Tartary Buckwheat Flavonoids and Amphibian Peptides. Adv. Mater. Res..

[39] Zhou X.-L., Chen Z.-D., Zhou Y.-M., Shi R.-H., Li Z.-J. (2019). The Effect of Tartary Buckwheat Flavonoids in Inhibiting the Proliferation of MGC80-3 Cells during Seed Germination. Molecules.

[40] Li Y., Duan S., Jia H., Bai C., Zhang L., Wang Z. (2014). Flavonoids from tartary buckwheat induce G2/M cell cycle arrest and apoptosis in human hepatoma HepG2 cells. Acta Biochim. Biophys. Sin..

[41] Liu Z., Ishikawa W., Huang X., Tomotake H., Kayashita J., Watanabe H., Kato N. (2001). A buckwheat protein product suppresses 1,2-dimethylhydrazine-induced colon carcinogenesis in rats by reducing cell proliferation. J. Nutr..

[42] Jiang L., Xie M., Chen G., Qiao J., Zhang H., Zeng X. (2020). Phenolics and Carbohydrates in Buckwheat Honey Regulate the Human Intestinal Microbiota. Evid. Based Complement. Altern. Med..

[43] Amelchanka S., Kreuzer M., Leiber F. (2010). Utility of buckwheat (*Fagopyrum esculentum* Moench) as feed: Effects of forage and grain on in vitro ruminal fermentation and performance of dairy cows. Anim. Feed Sci. Technol..

[44] Zhou X.-L., Yan B.-B., Xiao Y., Zhou Y.-M., Liu T.-Y. (2018). Tartary buckwheat protein prevented dyslipidemia in high-fat diet-fed mice associated with gut microbiota changes. Food Chem. Toxicol..

[45] Zhou Y., Zhao S., Jiang Y., Wei Y., Zhou X. (2019). Regulatory Function of Buckwheat-Resistant Starch Supplementation on Lipid Profile and Gut Microbiota in Mice Fed with a High-Fat Diet. J. Food Sci..

[46] Zhou Y., Wei Y., Yan B., Zhao S., Zhou X. (2020). Regulation of tartary buckwheat-resistant starch on intestinal microflora in mice fed with high-fat diet. Food Sci. Nutr..

[47] Huang Z.-R., Deng J.-C., Li Q.-Y., Cao Y.-J., Lin Y.-C., Bai W.-D., Liu B., Rao P.-F., Ni L., Lv X.-C. (2020). Protective Mechanism of Common Buckwheat (*Fagopyrum esculentum* Moench.) against Nonalcoholic Fatty Liver Disease Associated with Dyslipidemia in Mice Fed a High-Fat and High-Cholesterol Diet. J. Agric. Food Chem..

[48] Wu W., Li Z., Qin F., Qiu J. (2021). Anti-diabetic effects of the soluble dietary fiber from tartary buckwheat bran in diabetic mice and their potential mechanisms. Food Nutr. Res..

[49] Ren Y., Wu S., Xia Y., Huang J., Ye J., Xuan Z., Li P., Du B. (2021). Probiotic-fermented black tartary buckwheat alleviates hyperlipidemia and gut microbiota dysbiosis in rats fed with a high-fat diet. Food Funct..

[50] Peng L., Zhang Q., Zhang Y., Yao Z., Song P., Wei L., Zhao G., Yan Z. (2019). Effect of tartary buckwheat, rutin, and quercetin on lipid metabolism in rats during high dietary fat intake. Food Sci. Nutr..

[51] Fotschki B., Juśkiewicz J., Jurgoński A., Amarowicz R., Opyd P., Bez J., Muranyi I., Petersen I.L., Llopis M.L. (2020). Protein-Rich Flours from Quinoa and Buckwheat Favourably Affect the Growth Parameters, Intestinal Microbial Activity and Plasma Lipid Profile of Rats. Nutrients.

[52] Mu C., Ding N., Hao X., Zhao Y., Wang P., Zhao J., Ren Y., Zhang C., Zhang W., Xiang B. (2019). Effects of different proportion of buckwheat straw and corn straw on performance, rumen fermentation and rumen microbiota composition of fattening lambs. Small Rumin. Res..

[53] Cui K., Wang Q., Wang S., Diao Q., Zhang N. (2019). The Facilitating Effect of Tartary Buckwheat Flavonoids and *Lactobacillus plantarum* on the Growth Performance, Nutrient Digestibility, Antioxidant Capacity, and Fecal Microbiota of Weaned Piglets. Animals.

[54] Giménez-Bastida J.A., Zielinski H., Piskula M., Zielinska D., Szawara-Nowak D. (2017). Buckwheat bioactive compounds, their derived phenolic metabolites and their health benefits. Mol. Nutr. Food Res..

[55] Lee M.-S., Shin Y., Jung S., Kim S.-Y., Jo Y.-H., Kim C.-T., Yun M.-K., Lee S.-J., Sohn J., Yu H.-J. (2017). The Inhibitory Effect of Tartary Buckwheat Extracts on Adipogenesis and Inflammatory Response. Molecules.

[56] Lee C.-C., Shen S.-R., Lai Y.-J., Wu S.-C. (2013). Rutin and quercetin, bioactive compounds from tartary buckwheat, prevent liver inflammatory injury. Food Funct..

[57] Nam T.G., Lim T.-G., Lee B.H., Lim S., Kang H., Eom S.H., Yoo M., Jang H.W., Kim D.-O. (2017). Comparison of Anti-Inflammatory Effects of Flavonoid-Rich Common and Tartary Buckwheat Sprout Extracts in Lipopolysaccharide-Stimulated RAW 264.7 and Peritoneal Macrophages. Oxidative Med. Cell. Longev..

[58] Karki R., Park C.-H., Kim D.-W. (2013). Extract of buckwheat sprouts scavenges oxidation and inhibits pro-inflammatory mediators in lipopolysaccharide-stimulated macrophages (RAW264.7). J. Integr. Med..

[59] Choi S.Y., Choi J.Y., Lee J.M., Lee S., Cho E.J. (2015). Tartary buckwheat on nitric oxide-induced inflammation in RAW264.7 macrophage cells. Food Funct..

[60] Li F., Zhang X., Li Y., Lu K., Yin R., Ming J. (2017). Phenolics extracted from tartary (*Fagopyrum tartaricum* L. Gaerth) buckwheat bran exhibit antioxidant activity, and an antiproliferative effect on human breast cancer MDA-MB-231 cells through the p38/MAP kinase pathway. Food Funct..

[61] Manach C., Morand C., Demigné C., Texier O., Régérat F., Rémésy C. (1997). Bioavailability of rutin and quercetin in rats. FEBS Lett..

[62] Carbonaro M., Grant G. (2005). Absorption of Quercetin and Rutin in Rat Small Intestine. Ann. Nutr. Metab..

[63] Jaganath I.B., Mullen W., Edwards C.A., Crozier A. (2006). The relative contribution of the small and large intestine to the absorption and metabolism of rutin in man. Free Radic. Res..

[64] Willis A.D. (2019). Rarefaction, Alpha Diversity, and Statistics. Front. Microbiol..

[65] Anderson M.J., Crist T.O., Chase J.M., Vellend M., Inouye B.D., Freestone A.L., Sanders N.J., Cornell H.V., Comita L.S., Davies K.F. (2011). Navigating the multiple meanings of beta diversity: A roadmap for the practicing ecologist. Ecol. Lett..

[66] Beards E., Tuohy K., Gibson G. (2010). Bacterial, SCFA and gas profiles of a range of food ingredients following in vitro fermentation by human colonic microbiota. Anaerobe.

[67] Martin-Gallausiaux C., Marinelli L., Blottiere H.M., Larraufie P., Lapaque N. (2021). SCFA: Mechanisms and functional importance in the gut. Proc. Nutr. Soc..

[68] Blaak E.E., Canfora E.E., Theis S., Frost G., Groen A.K., Mithieux G., Nauta A., Scott K., Stahl B., Van Harsselaar J. (2020). Short chain fatty acids in human gut and metabolic health. Benef. Microbes.

[69] Takahashi Y., Ichikawa S., Aihara Y., Yokota S. (1998). Buckwheat allergy in 90,000 school children in Yokohama. Arerugi.

[70] Fok J.S., Kette F., Smith W.B., Smith A., Ahmadie A., Heddle R., Hissaria P. (2019). Buckwheat allergy in Australia. Intern. Med. J..

[71] Page M.J., McKenzie J.E., Bossuyt P.M., Boutron I., Hoffmann T.C., Mulrow C.D., Shamseer L., Tetzlaff J.M., Akl E.A., Brennan S.E. (2021). The PRISMA 2020 statement: An updated guideline for reporting systematic reviews. BMJ.

