# Supplementary material for: Systematic Review of Human and Animal Evidence on the Role of Buckwheat Consumption on Gastrointestinal Health
Source: Nutrients. 2022 Dec 20;15(1):1. doi: 10.3390/nu15010001 (PMC9823958; doi:10.3390/nu15010001)
Supplement: Supplementary file 1 [file nutrients-15-00001-s001.zip › nutrients-1992710-supplementary.pdf]

PRISMA 2020 Checklist

Supplemental Appendix SI. PRISMA checklist for the systematic review of human and animal evidence on the role of buckwheat consumption on gastrointestinal health.

Section and Topic	Item #	Checklist item	Location where item is reported
**TITLE**			
Title	1	Identify the report as a systematic review.	Line2
**ABSTRACT**			
Abstract	2	See the PRISMA 2020 for Abstracts checklist.	Line 22-36
**INTRODUCTION**			
Rationale	3	Describe the rationale for the review in the context of existing knowledge.	Line 40-59
Objectives	4	Provide an explicit statement of the objective(s) or question(s) the review addresses.	Line 65-71
**METHODS**			
Eligibility criteria	5	Specify the inclusion and exclusion criteria for the review and how studies were grouped for the syntheses.	Line 93-111
Information sources	6	Specify all databases, registers, websites, organisations, reference lists and other sources searched or consulted to identify studies. Specify the date when each source was last searched or consulted.	Line 75-82
Search strategy	7	Present the full search strategies for all databases, registers and websites, including any filters and limits used.	Supplement Appendix II
Selection process	8	Specify the methods used to decide whether a study met the inclusion criteria of the review, including how many reviewers screened each record and each report retrieved, whether they worked independently, and if applicable, details of automation tools used in the process.	Line 119-127
Data collection process	9	Specify the methods used to collect data from reports, including how many reviewers collected data from each report, whether they worked independently, any processes for obtaining or confirming data from study investigators, and if applicable, details of automation tools used in the process.	Line 119-127; Supplement Appendix II
Data items	10a	List and define all outcomes for which data were sought. Specify whether all results that were compatible with each outcome domain in each study were sought (e.g. for all measures, time points, analyses), and if not, the methods used to decide which results to collect.	Line 112-117; Supplement Appendix II
	10b	List and define all other variables for which data were sought (e.g. participant and intervention characteristics, funding sources). Describe any assumptions made about any missing or unclear information.	Line 112-117; Supplement Appendix II
Study risk of bias assessment	11	Specify the methods used to assess risk of bias in the included studies, including details of the tool(s) used, how many reviewers assessed each study and whether they worked independently, and if applicable, details of automation tools used in the process.	Line 119-127
Effect measures	12	Specify for each outcome the effect measure(s) (e.g. risk ratio, mean difference) used in the synthesis or presentation of results.	NA
Synthesis methods	13a	Describe the processes used to decide which studies were eligible for each synthesis (e.g. tabulating the study intervention characteristics and comparing against the planned groups for each synthesis (item #5)).	Line 45 (described in the study of Muka et al.
	13b	Describe any methods required to prepare the data for presentation or synthesis, such as handling of missing summary statistics, or data conversions.	Line 45 (described in the study of Muka et al.
	13c	Describe any methods used to tabulate or visually display results of individual studies and syntheses.	NA

Section and Topic	Item #	Checklist item	Location where item is reported
	13d	Describe any methods used to synthesize results and provide a rationale for the choice(s). If meta-analysis was performed, describe the model(s), method(s) to identify the presence and extent of statistical heterogeneity, and software package(s) used.	NA
	13e	Describe any methods used to explore possible causes of heterogeneity among study results (e.g. subgroup analysis, meta-regression).	NA
	13f	Describe any sensitivity analyses conducted to assess robustness of the synthesized results.	NA
Reporting bias assessment	14	Describe any methods used to assess risk of bias due to missing results in a synthesis (arising from reporting biases).	Line 119-127
Certainty assessment	15	Describe any methods used to assess certainty (or confidence) in the body of evidence for an outcome.	Line 119-127
**RESULTS**			
Study selection	16a	Describe the results of the search and selection process, from the number of records identified in the search to the number of studies included in the review, ideally using a flow diagram.	Line 146; Figure 1
	16b	Cite studies that might appear to meet the inclusion criteria, but which were excluded, and explain why they were excluded.	Line 146; Figure 1
Study characteristics	17	Cite each included study and present its characteristics.	Table 1-4
Risk of bias in studies	18	Present assessments of risk of bias for each included study.	Table 1-4
Results of individual studies	19	For all outcomes, present, for each study: (a) summary statistics for each group (where appropriate) and (b) an effect estimate and its precision (e.g. confidence/credible interval), ideally using structured tables or plots.	Table 1-4
Results of syntheses	20a	For each synthesis, briefly summarise the characteristics and risk of bias among contributing studies.	Line 129-371
	20b	Present results of all statistical syntheses conducted. If meta-analysis was done, present for each the summary estimate and its precision (e.g. confidence/credible interval) and measures of statistical heterogeneity. If comparing groups, describe the direction of the effect.	NA
	20c	Present results of all investigations of possible causes of heterogeneity among study results.	NA
	20d	Present results of all sensitivity analyses conducted to assess the robustness of the synthesized results.	NA
Reporting biases	21	Present assessments of risk of bias due to missing results (arising from reporting biases) for each synthesis assessed.	Line 364-370; Supplemental Table 1-3
Certainty of evidence	22	Present assessments of certainty (or confidence) in the body of evidence for each outcome assessed.	Line 364-370; Supplemental Table 1-3
**DISCUSSION**			
Discussion	23a	Provide a general interpretation of the results in the context of other evidence.	Line 374-380
	23b	Discuss any limitations of the evidence included in the review.	Line 462-473
	23c	Discuss any limitations of the review processes used.	Line 462-473
	23d	Discuss implications of the results for practice, policy, and future research.	Line 462-479
**OTHER INFORMATION**			
Registration and	24a	Provide registration information for the review, including register name and registration number, or state that the review was not registered.	Line 78-80

PRISMA 2020 Checklist

Section and Topic	Item #	Checklist item	Location where item is reported
protocol	24b	Indicate where the review protocol can be accessed, or state that a protocol was not prepared.	Line 78-80
	24c	Describe and explain any amendments to information provided at registration or in the protocol.	Line 78-80
Support	25	Describe sources of financial or non-financial support for the review, and the role of the funders or sponsors in the review.	Line 493-496
Competing interests	26	Declare any competing interests of review authors.	Line 503
Availability of data, code and other materials	27	Report which of the following are publicly available and where they can be found: template data collection forms; data extracted from included studies; data used for all analyses; analytic code; any other materials used in the review.	Line 78-80

From: Page MJ, McKenzie JE, Bossuyt PM, Boutron I, Hoffmann TC, Mulrow CD, et al. The PRISMA 2020 statement: an updated guideline for reporting systematic reviews. BMJ 2021;372:n71. doi: 10.1136/bmj.n71

For more information, visit: http://www.prisma-statement.org/

Supplemental Appendix SII. The study protocol of the systematic review of human and animal evidence on the role of buckwheat consumption on gastrointestinal health.

Systematic review of human and animal evidence on the role of buckwheat consumption on gastrointestinal health: a study protocol

1. Aim

The aim of current systematic review is to: (i) summarize human and animal evidence on the role of buckwheat on gastrointestinal health and microbiome and (ii) critically assess methodological quality of evidence, identify research gaps in literature, and provide directions for further research.

2. Literature search

Four online medical databases including EMBASE (Embase.com), MEDLINE via Ovid, Cochrane Central (Wiley) and Web of Science (Clarivate) were searched from date of inception until 17.01.2022. The Google Scholar was additionally search and the 200 most relevant references were downloaded using Publish or Perish software. The search included terms related to buckwheat and its scientific names and coupled with terms that relate to GI conditions, outcomes and symptoms. Search strategy can be found in Appendix I.

3. Study Selection Criteria

Condition/ domain being studied

- Healthy individuals/animals or individuals/animals with inflammatory bowel disease (IBD), irritable bowel syndrome (IBS), celiac disease (CeD) or those with gastrointestinal tumors and or any other disease of the gastrointestinal tract

Participants/population

- Human and animal study participants of any age

Study design

- Observational studies (exploring habitual buckwheat intake), intervention studies/randomized controlled trials (RCT) and non-randomized trials (exploring buckwheat supplementation or extracts originating from buckwheat) or pre-clinical studies using human material
- All animal, in vitro or in vivo studies
- No restrictions in sense of exposure or interventions or control groups across the studies

Exclusion criteria

• Letters to the editor
• Reviews
• Commentaries
• Conference abstracts

4. Study screening and Data extraction

The titles and abstracts will be independently evaluated by two reviewers and the full-texts will be assessed by two independent reviewers. In cases of disagreement, consensus will be reached by consulting a third reviewer. Data extraction will be done using a pre-defined data extraction form that included author and publication year, study design, population or sample characteristics, measurements or outcomes and other relevant data.

5. Methodological quality assessment

For the controlled, one arm non-randomized, and observational trials, the National Heart Lung and Blood Institute Quality Assessment Tool will be used. For the animal in vivo and in vitro studies, the Toxicological data Reliability Assessment Tool (ToxRTool) will be applied. Quality assessment is to be performed by two independent reviewers.

6. Data synthesis

Due to substantial heterogeneity across study designs and study populations (human and animal) we plan to provide a narrative synthesis of the evidence. We plan to do this by providing an overview of the evidence distinguishing between animal and human evidence and the quality of available studies. The narrative of the manuscript will be organized per study outcome (e.g., gastrointestinal symptoms, gut microbiome etc.) We plan to provide a graphical summary of the most important evidence to ease the interpretation of findings.

The complete search strategy for the systemic review on the role of buckwheat consumption on GI health

Embase,
('hemorrhoid'/de OR 'biliary tract disease'/exp OR 'flatulence'/exp OR 'eructation'/de OR 'peptic ulcer'/exp OR 'indigestion'/de OR 'heartburn'/de OR 'gastroesophageal reflux'/exp OR 'acid reflux'/de OR 'enteritis'/exp OR 'digestive system disease'/exp OR 'gut microbiome'/exp OR 'intestine flora'/exp OR 'feces microflora'/de OR 'bacterial count'/de OR 'dysbiosis'/de OR 'Bacteroidaceae'/exp OR 'microflora'/de OR 'microbiome'/exp OR 'microbial consortium'/de OR 'microbial diversity'/de OR 'gastrointestinal disease'/de OR 'gastrointestinal tract'/exp OR 'gastrointestinal motility'/exp OR 'intestine function'/exp OR 'gastrointestinal disease'/de OR 'gastrointestinal symptom'/exp OR 'digestive system inflammation'/exp OR 'enteropathy'/exp OR 'stomach disease'/exp OR 'bloating'/de OR 'abdominal pain'/exp OR 'intestine function disorder'/exp OR 'celiac disease'/de OR 'inflammatory bowel disease'/exp OR 'ulcerative colitis'/de OR 'colon tumor'/exp OR 'prebiotic agent'/de OR (enteric-bacteria* OR cystic-fibrosis OR gagging OR enteropath* OR enteritis OR acid-refl* OR microbi* OR microbe* OR coeliak* OR dysbios* OR dys-symbios* OR dysbacterios* OR disbacterios* OR ((enteric* OR alimentary-tract* OR bowel OR intestin* OR gastro-intestin* OR gastrointestin* OR gut OR colon* OR fecal OR feces) NEAR/3 (flora OR microflora* OR micro-flora* OR bacteri* OR microorganism* OR micro-organism* OR microbi*)) OR ((gastrointestin* OR gastro-intestin* OR intestin* OR bowel* OR colon* OR colorectal* OR mesocolon* OR gut OR GI OR celiac OR coeliac OR alimentary OR pancrea* OR gastri* OR stomach* OR prepyloric*) NEAR/3 (disease* OR disorder* OR carcin* OR cancer* OR tumor* OR tumour* OR neoplas* OR infection* OR syndrome* OR regulation* OR function* OR system* OR wellbeing OR well-being OR health OR miscomfort* OR pain OR dysplas* OR irrat* OR discolour* OR discolor* OR symptom* OR complain* OR discomf* OR distress* OR disturb* OR problem* OR side-effect* OR upset* OR toxic* OR inflamm* OR failure* OR fibrosis* OR lesion* OR deficien*)) OR ((esophagus OR oesophagus OR hepatobil* OR pancrea* OR alimentary-canal* OR alimentary-tract*) NEAR/3 (cancer* OR carcino* OR tumor* OR tumour*)) OR ((biliar*) NEAR/2 (tract*) NEAR/3 (disease* OR disorder* OR fibros* OR infect* OR inflamm* OR malform* OR pain OR tumour* OR tumor* OR neoplas* OR carcin* OR cancer*)) OR ((gallbladder OR cholecystopath*) NEAR/3 (diseas* OR disorder* OR pain OR tumour* OR tumor* OR neoplas* OR carcin* OR cancer* Or disfunct* OR dysfunct* OR polyp* OR dysmob* OR motil*)) OR ((intestinal OR gastrointestinal OR gastro-intestinal OR gastri*) NEAR/3 (tract* OR motilit* OR empt*)) OR IBD OR crohn* OR Terminal-Ileit* OR ((ulcera*) NEAR/6 (colit* OR colorec* OR procto*)) OR ((inflamm* OR irrita*) NEAR/3 (bowel* OR colon)) OR gastroenteropath* OR gastro-enteropath* OR enteropath* OR colitis OR digestive* OR bloating OR dyspep* OR ((abdomen OR abdominal) NEAR/3 (pain)) OR diarrh* OR constipat* OR obstipat* OR prebiotic* OR pre-biotic* OR peptic-ulcer* OR indigestion* OR pyrosis OR heartburn* OR heart-burn* GERD OR ((esophageal* OR gastroesoph* OR gastro-esoph* OR esophago* OR gastri* OR oesophago* OR oesophag* OR gastrooesophageal* OR gastro-oesophageal* OR duodenogastric* OR duodeno-gastric*) NEAR/3 (reflux* OR regurgit*)) OR barrett-esophag* OR barrett-oesophag* OR Zollinger-ellison* OR ((colon* OR intestin* OR jejunum OR peptic* OR rectum OR digesti* OR barrett* OR esophagus* OR stomach* OR stress OR gastro-duodenal* OR gastroduodenal* OR pepticum* OR gastri* OR gastro-intes* OR gastrointes*) NEAR/3 (ulcer* OR ulcus*)) OR gastroenteritis OR gastritis OR gastritides OR pancreatitis OR belching* OR ructus OR eructat* OR flatulen* OR flatus OR gastrointestinal-air* OR gastro-intestinal-air* OR cholelithias* OR gallstone* OR ((bile* OR gall*) NEAR/3 (stone*)) OR ((fecal OR feces OR defaecat* OR defecat* OR poo OR poop*) NEAR/3 (disorder* OR disease*)) OR hemorrhoid* OR haemorrhoid*):ab,ti,kw) **AND** ('fagopyrum'/exp OR (buckwheat* OR buck-wheat* OR fagopyrumesculent* OR ((fagopyrum* OR Polygonum*) NEAR/3 (escul* OR tatar* OR tarar* OR tartar* OR spp OR tartica*)) OR ((f) NEAR/2 (esculentum OR tataricum OR tartar OR tartarian OR tarticarium))):ab,ti,kw)

Medline
("Hemorrhoids"/ OR exp "Biliary Tract Diseases"/ OR "Flatulence"/ OR "Eructation"/ OR exp "Peptic Ulcer"/ OR "Dyspepsia"/ OR "Heartburn"/ OR exp "Gastroesophageal Reflux"/ OR exp "Enteritis"/ OR exp "Digestive System Diseases"/ OR "Gastrointestinal Microbiome"/ OR "Bacterial Load"/ OR "Dysbiosis"/ OR exp "Bacteroidaceae"/ OR "Microbiota"/ OR "Microbial Consortia"/ OR exp "Gastrointestinal Tract"/ OR exp "Gastrointestinal Motility"/ OR exp "Abdominal Pain"/ OR (enteric-bacteria* OR cystic-fibrosis OR gagging OR enteropath* OR enteritis OR acid-refl* OR microbi* OR microbe* OR coeliak* OR dysbios* OR dys-symbios* OR dysbacterios* OR disbacterios* OR ((enteric* OR alimentary-tract* OR bowel OR intestin* OR gastro-intestin* OR gastrointestin* OR gut OR colon* OR fecal OR feces) ADJ3 (flora OR microflora* OR micro-flora* OR bacteri* OR microorganism* OR micro-organism* OR microbi*)) OR ((gastrointestin* OR gastro-intestin* OR intestin* OR bowel* OR colon* OR colorectal* OR mesocolon* OR gut OR GI OR celiac OR coeliac OR alimentary OR pancrea* OR gastri* OR stomach* OR

prepyloric*) ADJ3 (disease* OR disorder* OR carcin* OR cancer* OR tumor* OR tumour* OR neoplas* OR infection* OR syndrome* OR regulation* OR function* OR system* OR wellbeing OR well-being OR health OR miscomfort* OR pain OR dysplas* OR irrat* OR discolour* OR discolor* OR symptom* OR complain* OR discomf* OR distress* OR disturb* OR problem* OR side-effect* OR upset* OR toxic* OR inflamm* OR failure* OR fibrosis* OR lesion* OR deficien*)) OR ((esophagus OR oesophagus OR hepatobil* OR pancrea* OR alimentary-canal* OR alimentary-tract*) ADJ3 (cancer* OR carcino* OR tumor* OR tumour*)) OR ((biliar*) ADJ2 (tract*) ADJ3 (disease* OR disorder* OR fibros* OR infect* OR inflamm* OR malform* OR pain OR tumour* OR tumor* OR neoplas* OR carcin* OR cancer*)) OR ((gallbladder OR cholecystopath*) ADJ3 (diseas* OR disorder* OR pain OR tumour* OR tumor* OR neoplas* OR carcin* OR cancer* Or disfunct* OR dysfunct* OR polyp* OR dysmob* OR motil*)) OR ((intestinal OR gastrointestinal OR gastro-intestinal OR gastri*) ADJ3 (tract* OR motilit* OR empt*)) OR IBD OR crohn* OR Terminal-Ileit* OR ((ulcera*) ADJ6 (colit* OR colorec* OR procto*)) OR ((inflamm* OR irrita*) ADJ3 (bowel* OR colon)) OR gastroenteropath* OR gastro-enteropath* OR enteropath* OR colitis OR digestive* OR bloating OR dyspep* OR ((abdomen OR abdominal) ADJ3 (pain)) OR diarrh* OR constipat* OR obstipat* OR prebiotic* OR pre-biotic* OR peptic-ulcer* OR indigestion* OR pyrosis OR heartburn* OR heart-burn* GERD OR ((esophageal* OR gastroesoph* OR gastro-esoph* OR esophago* OR gastri* OR oesophago* OR oesophag* OR gastrooesophageal* OR gastro-oesophageal* OR duodenogastric* OR duodeno-gastric*) ADJ3 (reflux* OR regurgit*)) OR barrett-esophag* OR barrett-oesophag* OR Zollinger-ellison* OR ((colon* OR intestin* OR jejunum OR peptic* OR rectum OR digesti* OR barrett* OR esophagus* OR stomach* OR stress OR gastro-duodenal* OR gastroduodenal* OR pepticum* OR gastri* OR gastro-intes* OR gastrointes*) ADJ3 (ulcer* OR ulcus*)) OR gastroenteritis OR gastritis OR gastritides OR pancreatitis OR belching* OR ructus OR eructat* OR flatulen* OR flatus OR gastrointestinal-air* OR gastro-intestinal-air* OR cholelithias* OR gallstone* OR ((bile* OR gall*) ADJ3 (stone*)) OR ((fecal OR feces OR defaecat* OR defecat* OR poo OR poop*) ADJ3 (disorder* OR disease*)) OR hemorrhoid* OR haemorrhoid*).ab,ti,kf) **AND** ("Fagopyrum"/ OR (buckwheat* OR buck-wheat* OR fagopyrumesculent* OR ((fagopyrum* OR Polygonum*) ADJ3 (escul* OR tatar* OR tarar* OR tartar* OR spp OR tartica*)) OR ((f) ADJ2 (esculentum OR tataricum OR tartar OR tartarian OR tarticarium))).ab,ti,kf.)

Web of Science

TS=(((enteric-bacteria* OR cystic-fibrosis OR gagging OR enteropath* OR enteritis OR acid-refl* OR microbi* OR microbe* OR coeliak* OR dysbios* OR dys-symbios* OR dysbacterios* OR disbacterios* OR ((enteric* OR alimentary-tract* OR bowel OR intestin* OR gastro-intestin* OR gastrointestin* OR gut OR colon* OR fecal OR feces) NEAR/2 (flora OR microflora* OR micro-flora* OR bacteri* OR microorganism* OR micro-organism* OR microbi*)) OR ((gastrointestin* OR gastro-intestin* OR intestin* OR bowel* OR colon* OR colorectal* OR mesocolon* OR gut OR GI OR celiac OR coeliac OR alimentary OR pancrea* OR gastri* OR stomach* OR prepyloric*) NEAR/2 (disease* OR disorder* OR carcin* OR cancer* OR tumor* OR tumour* OR neoplas* OR infection* OR syndrome* OR regulation* OR function* OR system* OR wellbeing OR well-being OR health OR miscomfort* OR pain OR dysplas* OR irrat* OR discolour* OR discolor* OR symptom* OR complain* OR discomf* OR distress* OR disturb* OR problem* OR side-effect* OR upset* OR toxic* OR inflamm* OR failure* OR fibrosis* OR lesion* OR deficien*)) OR ((esophagus OR oesophagus OR hepatobil* OR pancrea* OR alimentary-canal* OR alimentary-tract*) NEAR/2 (cancer* OR carcino* OR tumor* OR tumour*)) OR ((biliar*) NEAR/2 (tract*) NEAR/2 (disease* OR disorder* OR fibros* OR infect* OR inflamm* OR malform* OR pain OR tumour* OR tumor* OR neoplas* OR carcin* OR cancer*)) OR ((gallbladder OR cholecystopath*) NEAR/2 (diseas* OR disorder* OR pain OR tumour* OR tumor* OR neoplas* OR carcin* OR cancer* Or disfunct* OR dysfunct* OR polyp* OR dysmob* OR motil*)) OR ((intestinal OR gastrointestinal OR gastro-intestinal OR gastri*) NEAR/2 (tract* OR motilit* OR empt*)) OR IBD OR crohn* OR Terminal-Ileit* OR ((ulcera*) NEAR/5 (colit* OR colorec* OR procto*)) OR ((inflamm* OR irrita*) NEAR/2 (bowel* OR colon)) OR gastroenteropath* OR gastro-enteropath* OR enteropath* OR colitis OR digestive* OR bloating OR dyspep* OR ((abdomen OR abdominal) NEAR/2 (pain)) OR diarrh* OR constipat* OR obstipat* OR prebiotic* OR pre-biotic* OR peptic-ulcer* OR indigestion* OR pyrosis OR heartburn* OR heart-burn* GERD OR ((esophageal* OR gastroesoph* OR gastro-esoph* OR esophago* OR gastri* OR oesophago* OR oesophag* OR gastrooesophageal* OR gastro-oesophageal* OR duodenogastric* OR duodeno-gastric*) NEAR/2 (reflux* OR regurgit*)) OR barrett-esophag* OR barrett-oesophag* OR Zollinger-ellison* OR ((colon* OR intestin* OR jejunum OR peptic* OR rectum OR digesti* OR barrett* OR esophagus* OR stomach* OR stress OR gastro-duodenal* OR gastroduodenal* OR pepticum* OR gastri* OR gastro-intes* OR gastrointes*) NEAR/2 (ulcer* OR ulcus*)) OR gastroenteritis OR gastritis OR gastritides OR pancreatitis OR belching* OR ructus OR eructat* OR flatulen* OR flatus OR gastrointestinal-air* OR gastro-intestinal-air* OR cholelithias* OR gallstone* OR ((bile* OR gall*) NEAR/2 (stone*)) OR ((fecal OR feces OR defaecat* OR defecat* OR poo OR poop*) NEAR/2 (disorder* OR disease*)) OR hemorrhoid* OR haemorrhoid*)) **AND** ((buckwheat* OR buck-wheat* OR fagopyrumesculent* OR ((fagopyrum* OR Polygonum*) NEAR/2 (escul* OR tatar* OR tarar* OR tartar* OR spp OR tartica*)) OR ((f) NEAR/2 (esculentum OR tataricum OR tartar OR tartarian OR tarticarium)))))

Cochrane

((enteric NEXT bacteria* OR cystic NEXT fibrosis OR gagging OR enteropath* OR enteritis OR acid NEXT refl* OR microbi* OR microbe* OR coeliak* OR dysbios* OR dys NEXT symbios* OR dysbacterios* OR disbacterios* OR ((enteric* OR alimentary NEXT tract* OR bowel OR intestin* OR gastro NEXT intestin* OR gastrointestin* OR gut OR colon* OR fecal OR feces) NEAR/3 (flora OR microflora* OR micro NEXT flora* OR bacteri* OR microorganism* OR micro NEXT organism* OR microbi*)) OR ((gastrointestin* OR gastro NEXT intestin* OR intestin* OR bowel* OR colon* OR colorectal* OR mesocolon* OR gut OR GI OR celiac OR coeliac OR alimentary OR pancrea* OR gastri* OR stomach* OR prepyloric*) NEAR/3 (disease* OR disorder* OR carcin* OR cancer* OR tumor* OR tumour* OR neoplas* OR infection* OR syndrome* OR regulation* OR

function* OR system* OR wellbeing OR well NEXT being OR health OR miscomfort* OR pain OR dysplas* OR irrat* OR discolour* OR discolor* OR symptom* OR complain* OR discomf* OR distress* OR disturb* OR problem* OR side NEXT effect* OR upset* OR toxic* OR inflamm* OR failure* OR fibrosis* OR lesion* OR deficien*)) OR ((esophagus OR oesophagus OR hepatobil* OR pancrea* OR alimentary NEXT canal* OR alimentary NEXT tract*) NEAR/3 (cancer* OR carcino* OR tumor* OR tumour*)) OR ((biliar*) ADJ2 (tract*) NEAR/3 (disease* OR disorder* OR fibros* OR infect* OR inflamm* OR malform* OR pain OR tumour* OR tumor* OR neoplas* OR carcin* OR cancer*)) OR ((gallbladder OR cholecystopath*) NEAR/3 (diseas* OR disorder* OR pain OR tumour* OR tumor* OR neoplas* OR carcin* OR cancer* Or disfunct* OR dysfunct* OR polyp* OR dysmob* OR motil*)) OR ((intestinal OR gastrointestinal OR gastro NEXT intestinal OR gastri*) NEAR/3 (tract* OR motilit* OR empt*)) OR IBD OR crohn* OR Terminal NEXT Ileit* OR ((ulcera*) NEAR/6 (colit* OR colorec* OR procto*)) OR ((inflamm* OR irrita*) NEAR/3 (bowel* OR colon)) OR gastroenteropath* OR gastro NEXT enteropath* OR enteropath* OR colitis OR digestive* OR bloating OR dyspep* OR ((abdomen OR abdominal) NEAR/3 (pain)) OR diarrh* OR constipat* OR obstipat* OR prebiotic* OR pre NEXT biotic* OR peptic NEXT ulcer* OR indigestion* OR pyrosis OR heartburn* OR heart NEXT burn* GERD OR ((esophageal* OR gastroesoph* OR gastro NEXT esoph* OR esophago* OR gastri* OR oesophago* OR oesophag* OR gastrooesophageal* OR gastro NEXT oesophageal* OR duodenogastric* OR duodeno NEXT gastric*) NEAR/3 (reflux* OR regurgit*)) OR barrett NEXT esophag* OR barrett NEXT oesophag* OR Zollinger NEXT ellison* OR ((colon* OR intestin* OR jejunum OR peptic* OR rectum OR digesti* OR barrett* OR esophagus* OR stomach* OR stress OR gastro NEXT duodenal* OR gastroduodenal* OR pepticum* OR gastri* OR gastro NEXT intes* OR gastrointes*) NEAR/3 (ulcer* OR ulcus*)) OR gastroenteritis OR gastritis OR gastritides OR pancreatitis OR belching* OR ructus OR eructat* OR flatulen* OR flatus OR gastrointestinal NEXT air* OR gastro NEXT intestinal NEXT air* OR cholelithias* OR gallstone* OR ((bile* OR gall*) NEAR/3 (stone*)) OR ((fecal OR feces OR defaecat* OR defecat* OR poo OR poop*) NEAR/3 (disorder* OR disease*)) OR hemorrhoid* OR haemorrhoid*):ab,ti,kw) **AND** ((buckwheat* OR buck-wheat* OR fagopyrumesculent* OR ((fagopyrum* OR Polygonum*) NEAR/3 (escul* OR tatar* OR tarar* OR tartar* OR spp OR tartica*)) OR ((f) NEAR/2 (esculentum OR tataricum OR tartar OR tartarian OR tarticarium))):ab,ti,kw)

Google Scholar

Buckwheat|buck-wheat| fagopyrumesculent|"fagopyrum esculentum" intestine|gut|gastrointestinal|celiac|microflora|"colon cancer"|"inflammatory bowel disease"|IBD|microbiome|prebiotic|digestive

Supplementary Table S1. Quality assessment of controlled intervention studies in the review of the role of buckwheat consumption on GI health using the National Heart Lung and Blood Institute Quality Assessment Tool.

Lead Author, Publication (Supplemental Reference); Year	Study Design	1	2	3	4	5	6	7	8	9	10	11	12	13	14	Risk of bias (Total score)[1]
Dinu, 2017 [23]; Italy	Randomised crossover trial	Y	NR	N	N	N	Y	NR	NR	NR	Y	Y	N	Y	NR	Moderate (5)
De Francischi, 1994 [24]; Brazil	Parallel	N	NR	NR	NR	NR	NR	N	NR	NR	NR	Y	N	N	NR	High (1)

Abbreviations: CD, cannot determine; NA, not applicable; NR, not reported
[1]Risk of bias rating (Low (75-100%), Moderate (25-75%), or High (0-25%))

Criteria used to assess risk of bias of controlled clinical trials:

1. Was the study described as randomized, a randomized trial, a randomized clinical trial, or an RCT?
2. Was the method of randomization adequate (i.e., use of randomly generated assignment)?
3. Was the treatment allocation concealed (so that assignments could not be predicted)?
4. Were study participants and providers blinded to treatment group assignment?
5. Were the people assessing the outcomes blinded to the participants' group assignments?
6. Were the groups similar at baseline on important characteristics that could affect outcomes (e.g., demographics, risk factors, co-morbid conditions)?
7. Was the overall drop-out rate from the study at endpoint 20% or lower of the number allocated to treatment?

8. Was the differential drop-out rate (between treatment groups) at endpoint 15 percentage points or lower?

9. Was there high adherence to the intervention protocols for each treatment group?
10. Were other interventions avoided or similar in the groups (e.g., similar background treatments)?
11. Were outcomes assessed using valid and reliable measures, implemented consistently across all study participants?

12. Did the authors report that the sample size was sufficiently large to be able to detect a difference in the main outcome between groups with at least 80% power?
13. Were outcomes reported or subgroups analyzed prespecified (i.e., identified before analyses were conducted)?

14. Were all randomized participants analyzed in the group to which they were originally assigned, i.e., did they use an intention-to-treat analysis?

Criteria used to assess risk of bias of pre-post study without control group:

1. Was the study question or objective clearly stated?
2. Were eligibility/selection criteria for the study population prespecified and clearly described?
3. Were the participants in the study representative of those who would be eligible for the test/service/intervention in the general or clinical population of interest?
4. Were all eligible participants that met the prespecified entry criteria enrolled?
5. Was the sample size sufficiently large to provide confidence in the findings?
6. Was the test/service/intervention clearly described and delivered consistently across the study population?

7. Were the outcome measures prespecified, clearly defined, valid, reliable, and assessed consistently across all study participants?

8. Were the people assessing the outcomes blinded to the participants' exposures/interventions?
9. Was the loss to follow-up after baseline 20% or less? Were those lost to follow-up accounted for in the analysis?

10. Did the statistical methods examine changes in outcome measures from before to after the intervention? Were statistical tests done that provided p values for the pre-to-post changes?
11. Were outcome measures of interest taken multiple times before the intervention and multiple times after the intervention (i.e., did they use an interrupted time-series design)?
12. If the intervention was conducted at a group level (e.g., a whole hospital, a community, etc.) did the statistical analysis take into account the use of individual-level data to determine effects at the group level?

Supplementary Table S2. Quality assessment of observational studies in the review of the role of buckwheat consumption on GI health using the National Heart Lung and Blood Institute Quality Assessment Tool.

Lead Author, Publication (Supplemental Reference); Year	Study Design	1	2	3	4	5	6	7	8	9	10	11	12	13	14	Risk of bias (Total score)[1]
Zheng, 2015 [25]; Japan	Cross-sectional	Y	Y	Y	Y	N	N	Y	Y	Y	N	Y	N	Y	Y	Moderate (10)
Pilipenko, 2019 [26]; Pilipenko 2019 [27]; Russia	Cohort	Y	Y	CD	NR	NR	N	N	Y	Y	Y	Y	NR	Y	N	Moderate (7)

Abbreviations: CD, cannot determine; NA, not applicable; NR, not reported

[1]Risk of bias rating (Low (75-100%), Moderate (25-75%), or High (0-25%))

Criteria used to asses risk of bias of cohort/cross-sectional studies:

1. Was the research question or objective in this paper clearly stated?
2. Was the study population clearly specified and defined?
3. Was the participation rate of eligible persons at least 50%?
4. Were all the subjects selected or recruited from the same or similar populations (including the same time period)? Were inclusion and exclusion criteria for being in the study prespecified and applied uniformly to all participants?
5. Was a sample size justification, power description, or variance and effect estimates provided?
6. For the analyses in this paper, were the exposure(s) of interest measured prior to the outcome(s) being measured?
7. Was the timeframe sufficient so that one could reasonably expect to see an association between exposure and outcome if it existed?
8. For exposures that can vary in amount or level, did the study examine different levels of the exposure as related to the outcome (e.g., categories of exposure, or exposure measured as continuous variable)?
9. Were the exposure measures (independent variables) clearly defined, valid, reliable, and implemented consistently across all study participants?
10. Was the exposure(s) assessed more than once over time?
11. Were the outcome measures (dependent variables) clearly defined, valid, reliable, and implemented consistently across all study participants?
12. Were the outcome assessors blinded to the exposure status of participants?
13. Was loss to follow-up after baseline 20% or less?
14. Were key potential confounding variables measured and adjusted statistically for their impact on the relationship between exposure(s) and outcome(s)?

Criteria used to asses risk of bias of case-control studies:

1. Was the research question or objective in this paper clearly stated and appropriate?
2. Was the study population clearly specified and defined?
3. Did the authors include a sample size justification?
4. Were controls selected or recruited from the same or similar population that gave rise to the cases (including the same timeframe)?
5. Were the definitions, inclusion and exclusion criteria, algorithms or processes used to identify or select cases and controls valid, reliable, and implemented consistently across all study participants?
6. Were the cases clearly defined and differentiated from controls?
7. If less than 100 percent of eligible cases and/or controls were selected for the study, were the cases and/or controls randomly selected from those eligible?
8. Was there use of concurrent controls?
9. Were the investigators able to confirm that the exposure/risk occurred prior to the development of the condition or event that defined a participant as a case?
10. Were the measures of exposure/risk clearly defined, valid, reliable, and implemented consistently (including the same time period) across all study participants?
11. Were the assessors of exposure/risk blinded to the case or control status of participants?
12. Were key potential confounding variables measured and adjusted statistically in the analyses? If matching was used, did the investigators account for matching during study analysis?

Supplementary Table S3. Quality assessment of *in vitro* and animal *in vivo* studies in the review of the role of buckwheat consumption on GI health using the ToxRTool.

Author, Publication (Supplemental Reference); Year	Test substance identification (4)	Test system characterisation (3)	Study design description (6)	Study results documentation (3)	Plausibility of study design and data (2)	Total	Risk of Bias[1]
Zhou, 2013 [38]; China	4	3	4	2	2	15	Reliable w/o restrictions
Liu, 2021 [7]; China	4	3	6	3	2	18	Reliable w/o restrictions
Liu, 2001 [41]; Japan	1	3	4	2	2	12	Reliable with restrictions
Mu, 2019 [52]; China	1	3	6	3	2	15	Reliable w/o restrictions
Zhou, 2019 [34]; China	2	3	6	3	2	16	Reliable w/o restrictions
Zhu, 2020 [33]; China	2	3	5	3	2	15	Reliable w/o restrictions
Zhou, 2019 [45]; China	2	3	6	3	2	16	Reliable w/o restrictions
Ren, 2021 [49]; China	2	3	6	3	2	16	Reliable w/o restrictions
Zhou, 2018 [44]; China	2	3	5	3	2	15	Reliable w/o restrictions
Peng, 2019 [50]; China	1	3	6	3	2	16	Reliable w/o restrictions
Wu, 2021 [48]; China	1	3	6	3	2	16	Reliable w/o restrictions
Galina, 2020 [31]; Latvia	2	3	6	3	2	16	Reliable w/o restrictions
Li, 2020 [32]; China	2	3	6	3	2	16	Reliable w/o restrictions
Dziedric, 2018 [35]; Poland	4	3	4	2	1	14	Reliable with restrictions
Swiatecka, 2013 [36]; Poland	4	3	4	2	1	14	Reliable with restrictions
Ishii, 2008 [29]; Japan	4	3	3	3	2	15	Reliable w/o restrictions
Afroz, 2016 [30]; Japan	3	1	3	2	2	11	Reliable with restrictions
Kim, 2007 [37]; Korea	2	3	4	2	0	11	Reliable with restrictions
Jiang, 2020 [42]; China	4	2	6	2	2	16	Reliable w/o restrictions
Amelchanka, 2010 [43]; Switzerland	3	2	4	2	1	12	Reliable with restrictions
Huang, 2020 [47]; China	4	3	6	3	2	18	Reliable w/o restrictions
Fotschki, 2020 [51]; Poland	4	3	6	3	2	18	Reliable w/o restrictions
Gimenez-Bastida, 2020 [28]; USA	4	3	6	3	1	17	Reliable w/o restrictions
Cui, 2019 [53]; China	2	3	6	3	2	16	Reliable w/o restrictions
Zhou, 2020 [46]; China	2	3	6	3	2	16	Reliable w/o restrictions
Zhou, 2019 [39]; China	3	3	6	3	2	17	Reliable w/o restrictions
Li, 2014 [40]; China	4	3	6	3	2	18	Reliable w/o restrictions

[1] Total 15-18 means reliable without restrictions; 11-14 means reliable with restrictions; <11 and not all key criteria met means generally unrealiable,

Supplemental Figure S1. Changes in communities comparing intervention with common buckwheat and control [32,33,47,52].

Author	Animal	Control	Actinobacteria	Bacteroidetes	Epsilonbacteraeota	Euryarchaeota	Firmicutes	Proteobacteria	Synergistetes	Verrucomicrobiota	Clostridiales	Christensellaceae	Lachnospiraceae	Rikenellaceae	Ruminococcaceae	Allobaculum	Blautia	Lactobacillus	Oscillospira	Streptococcus
			Phylum								Order	Family				Genus				
Li, 2020	Mice	High salt diet									↓		↓	↑					↓	
Huang, 2020	Mice	High fat diet														↓	↑	↑		↑
Zhu, 2020	Mice	None	↓		↑			↑		↓										
Mu, 2019	Lambs	Regular diet		↑		↑	↓	↑	↓	↓		↓	↓	↑	↓					

Mice Lambs ↓ Decreased as compared to group without buckwheat ↑ Increased as compared to group without buckwheat

Supplemental Figure S2. Changes in communities comparing intervention with Tartary buckwheat and control [44,45,48-50,53]

Author	Animal	Control	Actinobacteria	Bacteroidetes	Euryarchaeota	Firmicutes	Proteobacteria	Synergistetes	Verrucomicrobiota	Christensellaceae	Lachnospiraceae	Rikenellaceae	Ruminococcaceae	Tannerellaceae	Akkermansia	Clostridium	Bifidobacterium	Blautia	Desulfovibrio	Enterococcus	Escherichia	Lactobacillus	Staphylococcus	Streptococcus
			Phylum							Family					Genus									
Zhou, 2018	Mice	High fat diet		↓												↑	↑			↑	↓	↑		
Zhou, 2019	Mice	High fat diet		↓													↑			↑	↓	↑		
Ren, 2021	Rats	High fat diet	↑			↑	↑											↑					↑	↑
Peng, 2019	Rats	High fat diet		↑										↓	↑				↓					
Wu, 2019	Rats	High fat diet		↑	↑	↓	↑	↓	↓	↓	↓		↓											
Cui, 2019	Pig	Basal diet										↑			↑									

Mice Rats Pig ↓ Decreased as compared to group without buckwheat ↑ Increased as compared to group without buckwheat
